# Energy Metabolism and Nutritional Condition of Juvenile White Sharks (*Carcharodon carcharias*; Linnaeus, 1978)

**DOI:** 10.3390/biology15151256

**Published:** 2026-07-31

**Authors:** Rebecca S. Lipscombe, Bianca S. Rangel, Stephen Morris, Paul A. Butcher

**Affiliations:** 1 National Marine Science Centre, Faculty of Science and Engineering, Southern Cross University, Coffs Harbour, NSW 2450, Australia; 2 Departamento de Fisiologia, Instituto de Biociências, Universidade de São Paulo, Rua do Matão, 321, Cidade Universitária, São Paulo 05508-090, SP, Brazil; biarangel.sharks@gmail.com; 3 Instituto Vida no Oceano, Fernando de Noronha 53990-000, PE, Brazil; 4 NSW Department of Primary Industries and Regional Development, Wollongbar, NSW 2477, Australia; stephen.morris@dpi.nsw.gov.au; 5 NSW Department of Primary Industries and Regional Development, National Marine Science Centre, Coffs Harbour, NSW 2450, Australia; paul.butcher@dpi.nsw.gov.au

**Keywords:** fatty acids, marine predator, nutritional ecology, plasma metabolites

## Abstract

Examining the relationship between an animal’s diet and physiology is crucial to understanding their responses to environmental change. Dietary-induced physiological variation can influence key life history processes, including growth and reproduction, ultimately affecting individual fitness. Reported here are the plasma biochemical parameters related to energy storage, use and nutritional condition in juvenile white sharks (*Carcharodon carcharias*) in Eastern Australia. Specifically, we used plasma metabolites (triglyceride, cholesterol and β-hydroxybutyrate), thyroid hormone thyroxine and fatty acid profiles from 50 juvenile white sharks (152–340 cm total length) captured on SMART drumlines in Eastern Australia to examine blood biochemistry and nutritional ecology. Concentrations of triglycerides and β-hydroxybutyrate indicated high energy expenditure likely associated with the broad-scale migration of white sharks along the Australian coast, highlighting the importance of β-hydroxybutyrate as an energy source. Seasonal variation in the triglyceride/cholesterol and ω3/ω6 fatty acid ratio, in conjunction with lower docosahexaenoic acid, suggests that lower-quality prey is consumed during winter months, thereby influencing the nutritional condition of these sharks. Additionally, the positive correlation between thyroxine concentrations and water temperature suggests reduced metabolic activity during cooler months. Such fluxes in food quality and nutritional condition may affect juvenile white sharks’ growth and development rate and future reproductive success.

## Simple Summary

Large marine predators like white sharks travel long distances along the East Australian coast. Changes in seasons mean different prey is available, which can impact the nutrition and energy use of these sharks. Understanding these changes is important for a protected species whose growth and survival depends on a consistent supply of food. This study measured the fats and energy markers in juvenile white shark blood, which reflect feeding, energy storage and nutrition, to examine how their condition changes with the seasons. Blood samples were collected from sharks in Northern New South Wales during their migration. Sharks that arrived in autumn had the highest levels of fats in their blood, suggesting recent feeding on good-quality prey. Reduced energy reserves and fat levels were seen in winter, indicating a leaner period. Considerable differences were seen in individual sharks, likely reflecting when and what each had eaten. Blood measures change over days to weeks and capture recent feeding rather than a fixed yearly pattern. These results show that a small blood sample is a minimally invasive way to assess the feeding and condition of wild sharks and may be useful in tracking how they respond to changes in the environment.

## 1. Introduction

Examining the link between an animal’s nutritional requirements, diet and physiology is crucial to understanding its responses to changes in the ecological environment [1]. Alterations in foraging behaviour may be a response to nutritional requirements, varying prey availability or environmental factors (e.g., water temperature), resulting in adjustments of physiological markers. Assessing such changes is necessary to evaluate the degree of impact that environmental and anthropogenic-driven changes have on the processes that support biological functions. The application of ecophysiology is widely explored in studies on terrestrial predators and marine taxa under controlled experimental conditions [2]. However, for large marine predators, such studies remain limited owing to the logistical and technical constraints of non-lethal sample collection in wild animals.

Predators are assumed to forage on prey with high energy content to maximise energy intake, demonstrating little consideration for balancing nutrient content through prey selection, supporting the idea that the driving factor in prey selection is energy maximisation [3]. Energy-centric foraging strategies are crucial in supporting the metabolic demands of migratory species, and nutrient balancing may be a trade-off to sustain energy requirements. Yet, few studies exploring the biochemical parameters associated with prey quality and energetics exist for sharks, despite prey nutrient composition varying with season and habitat [4,5,6,7]. Understanding the nutritional ecology of top predators is crucial to addressing management and conservation issues arising from anthropogenic change.

Energy storage and metabolism facilitate essential activities and functions required for animal survival. Accumulation of energy stores assists in mitigating resource scarcity over spatial and temporal scales [8]. Large migratory shark species have high energy requirements [9,10], exploiting various resources over broad spatial and temporal scales, which is reflected in their energy storage [11]. Body condition metrics, most often a mass–length relationship, are used to estimate an animal’s energy stores [12] but may not always accurately reflect the health or energetic state of an animal [13]. Therefore, using more direct measures, such as biomarkers associated with energy metabolism, can elucidate an accurate physiological state of the animal.

Plasma lipids, metabolites and hormones offer insight into energy allocation and expenditure in sharks [7,12]. Triglycerides and cholesterol are primary indicators of lipid reserves and are often elevated during periods of energy surplus [10], while β-hydroxybutyrate, a ketone body, reflects lipid mobilisation during fasting or high energy demand [14]. Total protein relates to nutritional status and muscle catabolism [15], whereas thyroxine, a thyroid hormone, plays a central role in regulating metabolic rate and is influenced by environmental temperature and physiological state [16]. Unlike other taxa, the unique energy metabolism of sharks results in a reliance on ketone bodies and amino acids for energy transport, with the storage and synthesis of lipids occurring in the liver and found in lower levels in plasma [17,18]. Triglycerides, cholesterol and free fatty acids are commonly used to study metabolism because of their rapid response to changes in feeding [12,19]. Similarly, ketone body β-hydroxybutyrate is a primary fuel used in aerobic metabolism and can indicate variation in muscular activity or mobilisation of lipids during periods of starvation [14]. Fatty acids play a key role in shark physiology and are useful as nutritional indicators [16,20]. Polyunsaturated fatty acids DHA, EPA and ARA, grouped as the essential fatty acids, are particularly important and required for growth, reproduction and cellular processes [21,22]. Additionally, the inability to synthesise these fatty acids de novo means they can only be obtained through dietary sources [23], which can be useful to explore prey quality and short-term impacts of diet on nutritional condition, as well as tissue mobilisation associated with specific physiological or ecological conditions (e.g., ref. [2]). Using essential fatty acids and plasma metabolites, inferences can be made on nutritional condition and health [6,13]. Together, these biomarkers provide a more nuanced understanding of shark bioenergetics and nutritional condition than body condition indices alone.

On the east coast of Australia, juvenile white sharks (*Carcharodon carcharias*) move through various coastal habitats, from temperate to tropical regions [24,25]. Seasonal movement patterns in these sharks have been documented: sharks move over a broad latitudinal range (~45°) in a north–south direction with high occupancy of waters in Southern QLD and Northern NSW during winter and spring months [26]. Seasonal plasticity in diet has recently been demonstrated in these sharks using stable isotopes and fatty acids of muscle [27], where a higher proportion of essential fatty acids was found during spring and summer, suggesting prey quality or availability is affecting essential fatty acids required for growth and development. Changes in prey availability are thought to drive these temporal variations in diet, which have been reported in other elasmobranch studies [28,29], yet the physiological impacts of this variation have not been explored.

Recent studies on shark physiology using fatty acid profiles and plasma metabolites focus on smaller coastal species [6,7,16] or are conducted under controlled/captive environments; therefore, extrapolating results to larger wide-ranging species is difficult. For example, responses of plasma metabolites to feeding and fasting have been explored experimentally in dogfish [14] and Port Jackson sharks [2]. While seasonal, ontogenetic and reproductive variation in plasma lipids and ketone bodies were described in wild small-spotted catsharks [30], nurse sharks [12] and life and reproductive states in several coastal species [6,7,16]. There are few studies describing the plasma biochemistry of larger, migratory species. Earlier studies of white sharks focused on muscle tissue lipids and energy density profiles related to feeding ecology [10] and effects of capture duration [31], but not nutritional ecology or condition. The capacity for plasma metabolites and fatty acids to elucidate the nutritional state of white sharks has not been reported, and expanding on the physiological and ecological data of these highly migratory and vulnerable species is necessary for effective conservation and management.

Here, we present an exploratory study examining the short-term nutritional condition and biochemical parameters associated with energy metabolism using plasma fatty acid profiles and metabolic biomarkers in juvenile white sharks captured in sub-tropical Eastern Australia. We quantify plasma metabolites and examine the relationship among these parameters and we evaluate the influence of sex and season on their variability. To evaluate the biological and environmental factors that influence condition and energy storage, we examine the effect of sex and season on the variability in metabolites and plasma fatty acids. We hypothesised that seasonal changes in prey availability and quality influences the variation in the nutritional condition and energetics of juvenile white sharks, and this would be reflected differently across biomarkers according to their physiological roles. Based on triglycerides and cholesterol reflecting lipid reserves and the triglyceride/cholesterol ratio indicating nutritional condition, we expect higher ratios during seasons where higher-quality prey is available [27]. We predicted β-hydroxybutyrate concentrations would remain low if sharks had recently fed, due to increases observed in other species during fasting or resulting from lipid mobilisation. Variation in essential fatty acids is expected as these are acquired through diet and will likely reflect seasonal changes in prey in this region. Finally, as a regulator of metabolic rate, increases in thyroxine with water temperature were predicted, reflecting higher metabolic activity during warmer months.

## 2. Materials and Methods

### 2.1. Capture and Sampling

**Figure 1:**
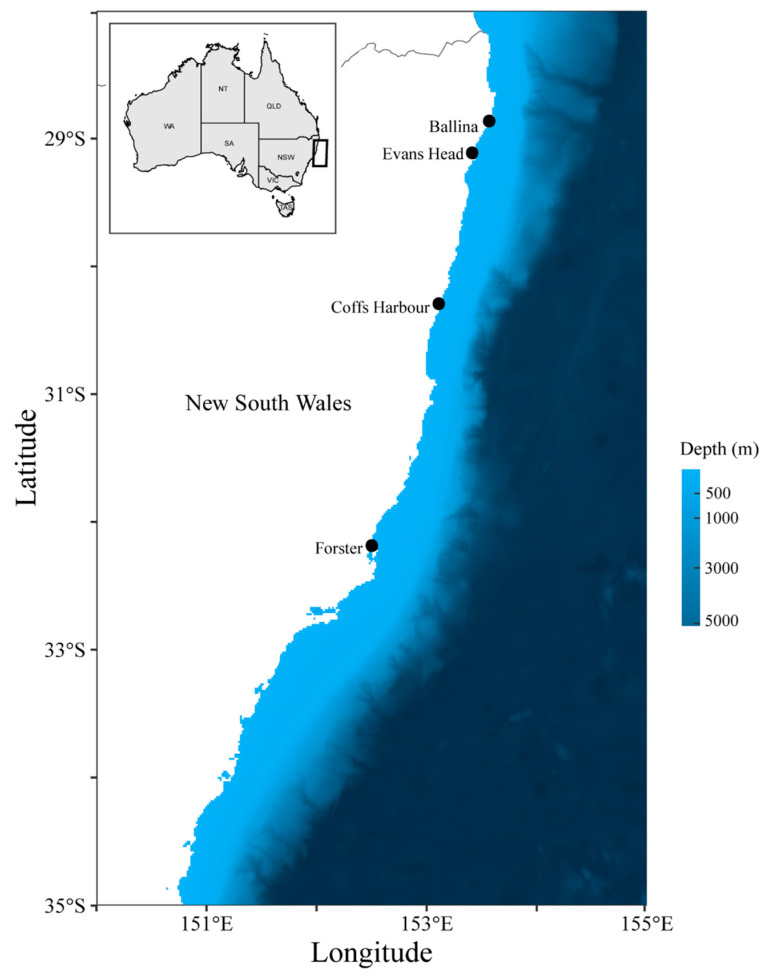
Map of the study area and sampling locations of white sharks (*Carcharodon carcharias*) in New South Wales, Australia. Inset shows the location of the study area within Australia.

Blood samples were collected from white sharks between 2016 and 2022 at four locations between Evans Head (29.1189° S, 153.4315° E) and Forster (32.1796° S, 152.5118° E), NSW, Australia (Figure 1; Table S1). Sampling was opportunistic as sharks were captured as part of the NSW Government Shark Management Program, using SMART (Shark-Management-Alert-in-Real-Time, Marine Instruments, Pontevedra, Spain) drumlines (see [32,33] for gear configuration). Capture on SMART drumlines has been found to have minimal impact on the blood chemistry and plasma fatty acids of white sharks [31,32]. Therefore, the effect of capture on the plasma metabolites and fatty acids used here was considered insignificant. Sharks were secured to the vessel upon capture via a cross-pectoral fin and tail rope. Total length (TL) was measured to the nearest cm and the presence or absence of external reproductive organs determined sex. Whole blood (~15 mL) was collected from the caudal vein using a 90 mm 18-gauge needle fitted to a 20 mL syringe. Blood was immediately transferred to 8 mL lithium heparin tubes (BD Vacutainer) and kept on ice until being centrifuged at 1534× *g* for 3 min. Separated plasma was removed and transferred to 2 mL vials. Depending on the collection location, samples were frozen at −18 °C in the field and later moved to −80 °C upon return to the laboratory or frozen immediately at −80 °C.

To examine the nutritional biomarkers, 22 plasma samples from white sharks (14 female: 8 male) captured in 2021 and 2022 were analysed by IDEXX laboratories (Brisbane, Australia) for concentrations of total protein (TP, grams per litre, g L^−1^), cholesterol (CHOL, millimoles per litre, mmol L^−1^), triglycerides (TAGs, millimoles per litre, mmol L^−1^), β-hydroxybutyrate (β-HB), millimoles per litre, mmol L^−1^) and thyroxine (T_4_, nanomoles per litre, nmol L^−1^).

### 2.2. Lipid Extraction

Plasma samples from 39 white sharks (26 female; 13 male) captured between 2016 and 2021 were lipid extracted before fatty acid analysis [34,35]. Briefly, plasma was lyophilised (1.2 ± SD 0.78 g) for 48 h (Alpha LD14 plus freeze dryer) and homogenised (Qiagen TissueLyser LT, Hilder, Germany) for 2 min at 50 Hz. Lipids were extracted from the lyophilised plasma by employing a modified Bligh and Dyer [36] method using 3.8 mL of dichloromethane:methanol:Milli-Q (1:2:0.8 mL) solution for approximately 18 h. The supernatant was transferred into a glass tube after centrifuging at 1534× *g* for 2 min. The polar and non-polar phases were separated using 1 mL each of dichloromethane and 0.9% NaCl saline solution and centrifuged at 1534× *g* for 2 min. The lower non-polar layer was transferred to a 2 mL vial and dried under N_2_ to a constant weight. Total lipid extract was resuspended in 1.5 mL of dichloromethane and stored at −80 °C for subsequent fatty acid analysis.

### 2.3. Fatty Acid Analysis

Fatty acid analysis of plasma was performed following a modified Bligh and Dyer [36] trans-methylation procedure, described in Meyer et al. [37], whereby an aliquot of the total lipid extract was trans-methylated using 3 mL of dichloromethane:methanol:hydrochloric acid (10:10:1 *v*/*v*/*v*) for 2 h at 80 °C. After cooling to room temperature, 1 mL of Milli-Q was added. The resulting FAMEs were extracted from the methylating solvent using three washes of 1.8 mL of hexane:dichloromethane (4:1 *v*/*v*) and centrifuged at 1534× *g* for 5 min. FAMEs contained in the upper solvent layer were collected in a 2 mL glass vial, dried under N_2_ gas and suspended in 1 mL of dichloromethane. Individual fatty acids were identified and quantified through GC. Peak separation of fatty acids was attained using an Agilent Technologies 7890B GC (Palo Alto, CA, USA) with an Equity-1 fused silica capillary column (15 mm × 0.1 mm internal diameter and 0.1 mm film thickness), a flame ionisation detector, a splitless injector and an Agilent Technologies 7683B Series auto-sampler. Samples were injected in splitless mode and transported by helium gas at 120 °C. The temperature was raised to 270 °C at a rate of 10 °C per min and then to 310 °C at a rate of 5 °C per min. We quantified fatty acid peaks using Agilent Technologies ChemStation software (C.01.09 SR2, Palo Alto, CA, USA) and confirmed identities using a Finnigan Thermoquest DSQ GC-MS system (Austin, TX, USA). Fatty acid chromatogram peak areas were converted to percentages of total area.

### 2.4. Nutritional Condition Indicators

Concentrations of triglyceride and cholesterol were used as indices of nutritional condition, in addition to essential fatty acid proportions and ratios of triglyceride/cholesterol [19,38] and ω3/ω6 [22,39].

### 2.5. Statistical Analyses

Empirical relationships between plasma concentrations of total proteins, triglycerides, cholesterol, β-hydroxybutyrate, thyroxine and water temperature were examined by calculating Pearson’s correlation coefficient (r) and testing the hypothesis *r* = 0 via t-test. Variation in metabolites was assessed in response to sex and the winter and spring seasons (winter: 3 males, 5 females and spring: 5 males, 9 females) via analysis of variance (ANOVA). There was insufficient data to include the effect of sampling in summer (1 female) and autumn (1 male) in the metabolite analysis. Variation in fatty acids was assessed in response to sex and the summer, autumn, winter and spring seasons via analysis of variance. Of the 35 fatty acids detected, those with averages > 0.1% were statistically analysed (see [27]). Residuals of all ANOVA and linear models were assessed for normality (visually via normal quantile plots and formally via the Shapiro–Wilk test) and for homogeneity of variance (Levene’s test). Residuals for ARA, PUFA and PUFAω6 departed from normality while variances were homogeneous, and these traits were therefore assessed using the non-parametric Kruskal–Wallis test. ARA/EPA met both assumptions and was assessed using an ANOVA test. Post hoc comparison of mean responses for traits meeting ANOVA assumptions was conducted using Tukey’s Honest Significant Difference. Effect sizes (η^2^) with 95% confidence intervals were calculated for seasonal comparisons to accompany the reported *p*-values. An exploratory principal component analysis was performed on the plasma metabolites (triglyceride, cholesterol, β-hydroxybutyrate and thyroxine) for the 22 individuals to assess whether distinct metabolite groups were formed; variables were centred to mean zero and scaled to unit standard deviation prior to analysis. All analyses were conducted in the R environment v4.3.1 [40], and statistical significance was defined at *p* < 0.05.

## 3. Results

Fifty white sharks were sampled in this study, with metabolite analysis completed on 22 individuals and fatty acid analysis on 39 individuals. Eleven individuals underwent both metabolite and fatty acid analysis. Sharks ranged in size from 182–340 cm TL (mean ± SD of 238 ± 41 cm; plasma metabolites) to 152–340 cm TL (250 cm ± 39 cm; fatty acids), suggesting all individuals were juveniles based on the life history characteristics described by Bruce and Bradford [25].

**Figure 2:**
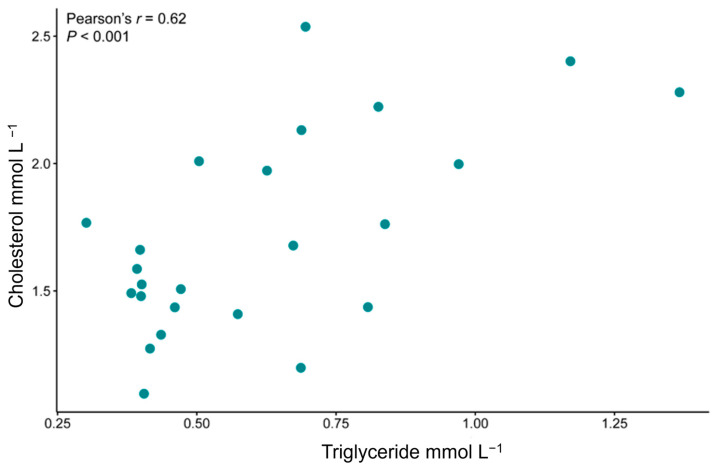
Pearson’s correlation between cholesterol mmol L^−1^ and triglycerides mmol L^−1^ from white shark plasma.

**Figure 3:**
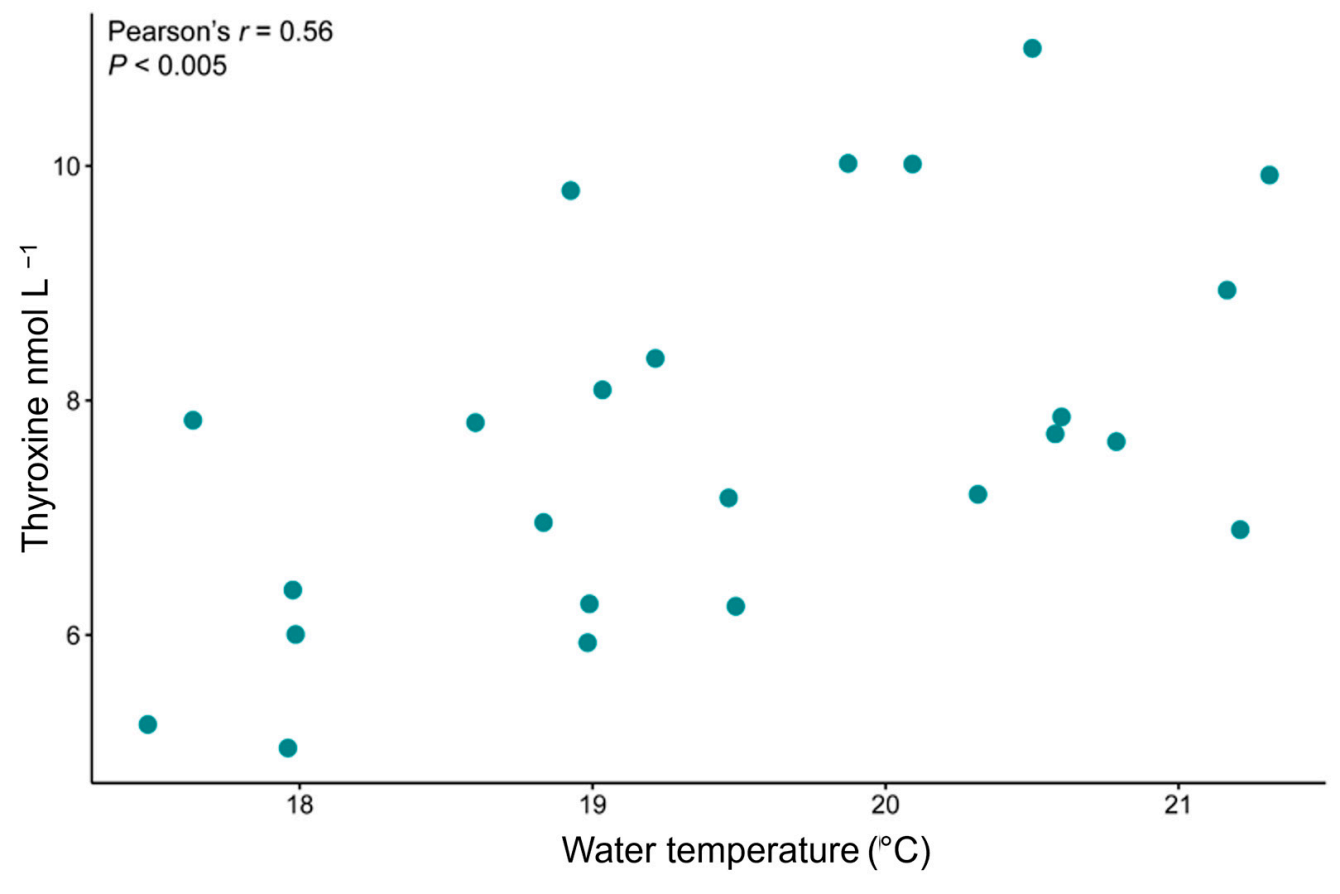
Pearson’s correlation between white shark plasma thyroxine nmol L^−1^ concentration and water temperature (°C).

**Figure 4:**
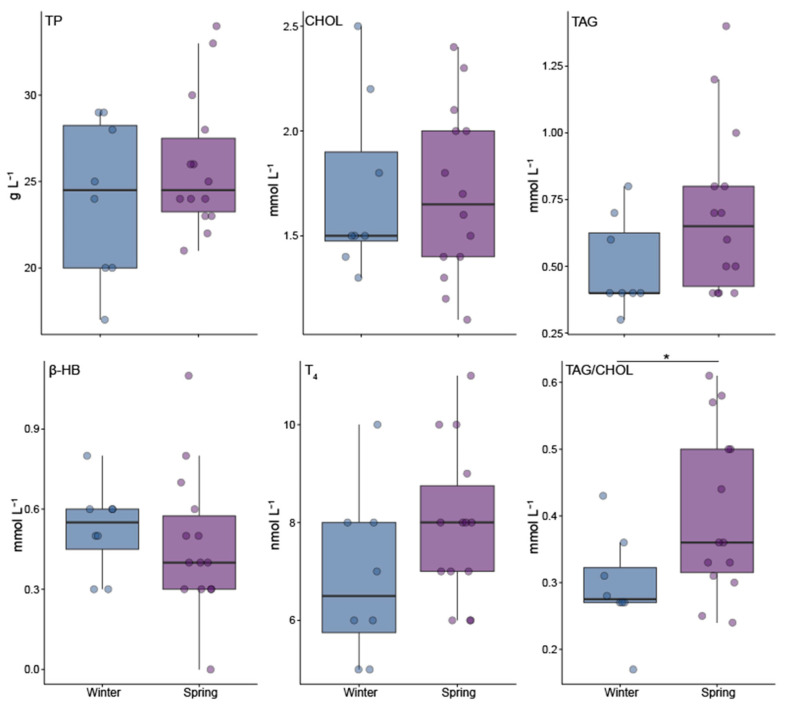
Observed seasonal variation in white shark plasma concentrations of total protein (TP), cholesterol (CHOL), triglycerides (TAGs), β-hydroxybutyrate (β-HB), thyroxine (T_4_) and triglyceride/cholesterol ratio (TAG/CHOL). * Denotes seasons with significantly different means (* *p* < 0.05).

For the 22 sharks providing metabolite data, values ranged as follows, total proteins, 17.0–34.0 g L^−1^ (mean ± SE of 25.04 ± 4.07, SD), triglycerides, 0.3–1.4 mmol L^−1^ (0.63 ± 0.28 mmol L^−1^), cholesterol, 1.1–2.5 mmol L^−1^ (1.72 ± 0.39 mmol L^−1^), β-hydroxybutyrate, 0.0–1.1 mmol L^−1^ (0.49 ± 0.22 mmol L^−1^), thyroxine 5.0–11.0 nmol L^−1^ (7.67 ± 1.69 nmol L^−1^) and triglyceride/cholesterol ratio 0.17–0.61 mmol L^−1^ (0.36 ± 0.12 mmol L^−1^). Significant positive correlations between triglyceride and cholesterol (Pearson’s correlation *r* = 0.62, *p* < 0.001, Figure 2; Supplementary Table S1) and between thyroxine and water temperature (*r* = 0.56, *p* < 0.005, Figure 3) were detected. Exploratory principal component analysis of the metabolites revealed no distinct groupings among individuals; the first two components explained 71% of the variance (47% and 24%; Figure S1). Significant differences in the mean triglyceride/cholesterol ratio between winter and spring were noted (one-way ANOVA *p* < 0.05, η^2^ = 0.20 CI [0.00, 0.48]; Figure 4), namely, higher spring concentrations than winter. No significant effects of shark sex on mean metabolite concentrations were detected (ANOVA; Supplementary Table S2).

**Figure 5:**
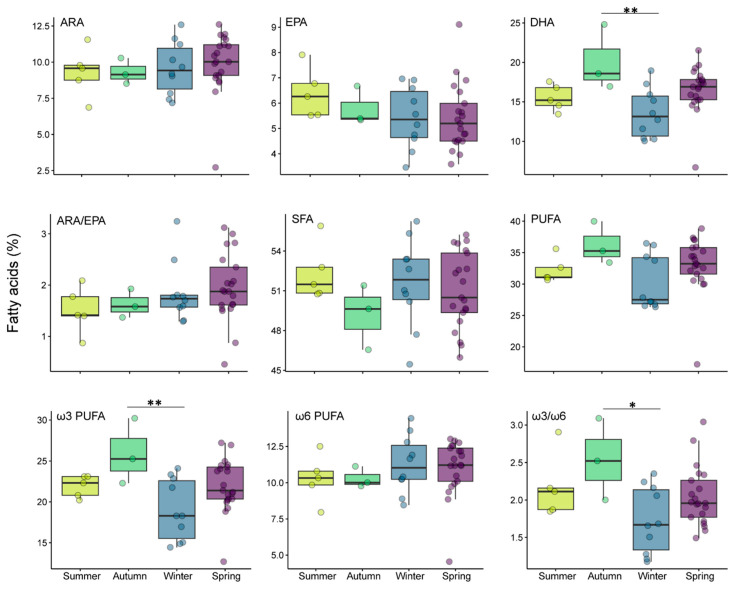
Observed seasonal variation in nutritionally important fatty acids in white shark plasma (*n* = 39). The line and asterisk denote groups with significantly different means (** *p* < 0.01, * *p* < 0.05).

Blood plasma comprised mainly saturated fatty acids (SFAs; 52% ± 0.9), followed by polyunsaturated fatty acids (PUFAs; 36% ± 1.2), which consisted of a high proportion of essential fatty acid DHA (16% ± 3.3). The ANOVA test indicated significant differences in the mean proportions of DHA, ω3 PUFA and the ratio of ω3/ω6 within season. Post hoc analysis (Tukey’s test) revealed that these fatty acids were significantly higher in autumn compared with winter (*p* < 0.05; Figure 5; Supplementary Table S3), with moderate-to-large but imprecisely estimated effect sizes (η^2^ = 0.22–0.28; 95% CI; Supplementary Table S3). No significant seasonal variation was shown in the remaining fatty acids assessed: ARA, PUFA and PUFAω6 (Kruskal–Wallis; ARA H = 1.82, *p* = 0.61; PUFA H = 5.84, *p* = 0.12; PUFAω6 H = 2.75, *p* = 0.43) and ARA/EPA (ANOVA F _3,35_ = 0.85, *p* = 0.48). Linear models did not detect an effect of TL on white shark plasma metabolite or fatty acid values (*p* > 0.05).

## 4. Discussion

Through analysis of plasma metabolites and fatty acid profiles, this study has elucidated new information on the nutritional condition and biochemical parameters related to diet and energetic state in juvenile white sharks. High individual variation in energy-related biochemical parameters was evident, suggesting individual differences in energetic needs and foraging. Triglyceride concentrations reflected a highly active species with high energy storage requirements to support physiological processes [10]. Seasonal variation in the triglyceride/cholesterol ratio suggests that the condition of white sharks may relate to changes in diet, previously reported by Lipscombe et al. [27]. This finding is further supported by the plasma fatty acids, whereby higher proportions of essential fatty acid DHA, ω3 PUFAs and ω3/ω6 were found in autumn compared with winter, suggesting that environmental factors linked to prey quality may affect the short-term nutritional condition of these sharks. These key findings are discussed below in relation to how fluctuations in prey availability across seasons may impact the nutritional status and energy dynamics of juvenile white sharks.

### 4.1. Energy Storage and Migration

The broad-scale migratory movement of white sharks in Eastern Australia (~1000–1500 km; [26,41]) coincides with seasonal changes, water temperature and prey species, with energetic variations seen here likely associated with migratory movement, variation in feeding occurrence and prey composition. Although not statistically significant, the concentration of triglycerides observed was lower in winter, coinciding with the recent movement from cooler waters in Southeastern Australia to Northern NSW. Energetic requirements to perform this broad-scale movement are high, and there is a reliance on stored energy in the liver in the form of lipids to sustain these migratory movements [9,10]. Average plasma triglyceride concentrations from migratory tiger sharks sampled in Florida and the Bahamas were lower (0.20 mmol L^−1^; [19]) compared with the white sharks in this study, suggesting there are energetic limitations of migration. The triglyceride concentrations reported here may be associated with recent migratory movements of white sharks in this region. However, linking physiological state to movement, specifically migration, is open to interpretation, whereas we have suggested the lower winter concentrations as a reflection of recent broad-scale movement. Gallagher et al. [42] suggests that elevated triglycerides in blue sharks (*Prionace glauca*) are indicative of energy stores reserved for migration, rather than recent feeding. Additional research on plasma and liver energy stores before migration may elucidate further information on the energetic state and cost of migration in white sharks.

Although elasmobranchs typically have a low capacity for cholesterol transport [43], a relationship between cholesterol and movement has been reported [44]. For example, in a preliminary study, Wosnick et al. [44] reported higher concentrations (4.2 mmol L^−1^) in juvenile tiger sharks before migration. Similar to triglyceride concentrations, the concentrations of cholesterol observed here suggest exhausted energy stores after broad-scale movement along the NSW coast. However, dietary origins cannot be excluded. Additionally, cholesterol is a precursor to steroid hormones [45], and as demands for this increase, cholesterol may change. Positive correlations of triglyceride and cholesterol have been observed in other elasmobranchs, most likely associated with variability in diet because of changing prey availability or seasonal changes in the nutritional profile of prey [12,30].

Concentrations of β-hydroxybutyrate in white shark plasma (0.0–1.1 mmol L^−1^; mean 0.49 mmol L^−1^) were higher than those reported in wild-sampled blacktip and nurse sharks (~0.02 mmol L^−1^; [7]), yet lower than the ~1 mmol L^−1^ recorded in the dogfish shark (*Squalus acanthias*) up to 60 h after feeding, following a decline from 5 mmol L^−1^ [14]. Earlier studies have linked this ketone body to physical exertion [30] and to fasting or starvation [14]. The variation in β-hydroxybutyrate concentrations reported here indicates sharks were in various states from recently fed to fasted. Lower concentrations in some individuals may indicate recent feeding, in contrast with others whose values are consistent with greater lipid mobilisation. These values plausibly reflect individual variation in feeding state at time of capture, consistent with between-individual variability evident among other energy-related metabolites. As capture (minimal on SMART drumlines; [32]), physical exertion and individual differences in metabolic rates can influence β-hydroxybutyrate levels, interpretation requires caution. Despite this, β-hydroxybutyrate was detected and elevated relative to several other elasmobranchs and remains consistent with its role as an aerobic substrate supporting the high activity levels associated with broad-scale migration in this species.

Water temperature was observed to be positively correlated with the metabolic thyroid hormone thyroxine, but no evidence of correlation with the other metabolites was found. Thyroid hormones have not been well studied in elasmobranchs, especially larger species. However, they play pivotal roles in regulating vertebrate metabolic and developmental processes [46] and are useful biomarkers to examine energetic variations. Of the few studies of these hormones in elasmobranchs, an association between higher water temperatures and higher concentrations of thyroid hormones was observed [47]. Additionally, the relationship between energetic changes and thyroxine has been investigated in marine mammals, in which decreased values were associated with fasting [48]. The lower concentrations observed here in white sharks associated with cooler water temperatures indicate a lower energy expenditure and likely increased reliance on internal energy stores. Conversely, as thyroxine increases with water temperature, the metabolic activity of white sharks increases and is supported through feeding. These results are further supported by the lower triglyceride/cholesterol ratios observed in winter. However, given the unusual metabolism of elasmobranchs, the regional endothermy of white sharks and the lack of understanding of how changing biotic and abiotic conditions influence this hormone, further research is required to improve understanding of the role of thyroid hormones in active shark species over larger temporal scales.

### 4.2. Seasonal Variation in Metabolites and Fatty Acids

The ratio of triglyceride to cholesterol is used as a metric of nutritional condition in many taxa, including elasmobranchs [12,49], in which higher ratios indicate better nutritional condition. We found that ratios of triglyceride/cholesterol in white shark plasma were higher during spring, coinciding with regular upwelling events in this region where chlorophyll *a* concentrations increase over the continental shelf [50,51]. Such increases directly support local food webs, serving as a proxy for prey abundance and foraging opportunities [52,53]. This finding suggests that the seasonal variation in prey is linked to the nutritional condition of juvenile white sharks. Increases in nutrient availability would be valuable to meet the metabolic requirements of migration and those required for growth. Additionally, the considerable inter-individual variation in the triglyceride/cholesterol ratio suggests that some individuals have a lower nutritional condition, which may be attributed to differences in foraging strategies or other biotic factors not measured in this study. This inter-individual variability, rather than a clear seasonal pattern, is consistent with the positive triglyceride/cholesterol relationship reflecting differences among individuals in diet and nutritional condition, likely arising from variation in prey availability and quality.

Seasonal variation in plasma essential fatty acids may be linked to feeding frequency, prey abundance or quality. Limited prey abundance or feeding frequency is unlikely because higher concentrations of β-hydroxybutyrate reflective of fasting were not evident [14]. Therefore, it is reasonable to suggest that prey quality influences the nutritional condition of white sharks. This finding is supported by a recent study [27] which used stable isotopes and fatty acids of muscle to demonstrate seasonal variations in white shark diet linked to a change in prey associated with season. Specifically, white shark muscle was depleted in essential fatty acids DHA, ARA and EPA during autumn and winter and enriched during spring and summer [27]. These changes were linked to a shift in prey with season, whereby higher proportions of mid-trophic level δ^13^C-depleted prey were consumed in winter. Notably, elevated proportions of DHA, ω3 PUFAs and ω3/ω6 ratio in plasma during the autumn, alongside a concurrent decline in muscle DHA [27], may indicate mobilisation of this omega-3 fatty acid from muscle to other tissues. Alternatively, DHA-enriched plasma in autumn may reflect recent feeding, and we acknowledge that our results cannot distinguish between these explanations. If mobilisation is occurring, it may compensate for seasonal changes in prey quality. Together, these patterns describe fluctuations across a temporal scale that coincides with the arrival of white sharks in Northern NSW after migration from cooler southern waters. Elevated plasma DHA and ω3 PUFAs in autumn sampled sharks may reflect recent feeding on nutrient-rich prey during migration, compared with decreased levels in winter sampled sharks, which, combined with the lower triglycerides, may be reflective of feeding on lower-quality prey. Rapid turnover rates of fatty acids in plasma suggest these proportions are reflective of recent dietary intake (days to weeks) and short-term mobilisation, rather than a fixed nutritional state through the season (e.g., ref. [2]). The extent of mobilisation is expected to vary among individuals with differing feeding history and physiological demands and remains consistent with the variability observed across the other plasma biomarkers reported here.

### 4.3. Limitations

The use of multiple physiological markers and fatty acids enables a greater understanding of the relationship between the diet and energetics of white sharks. However, we acknowledge there are limitations in this study due to opportunistic sampling, resulting in a small sample size for metabolite and thyroid hormone analyses, which were restricted to winter and spring. The lack of samples obtained during summer and autumn, when white shark presence and capture are low [33,54], may result in the inability to detect further seasonal patterns in energetic state and increase the risk of Type II error for non-significant comparisons. We therefore interpret non-significant results as inconclusive rather than as evidence of no effect and report effect sizes with 95% confidence intervals (Supplementary Table S3). Several seasonal comparisons indicated moderate to large effects, whose confidence intervals extended close to zero (e.g., ω3/ω6, η^2^ = 0.22, 95% CI [0.00, 0.42]; triglyceride/cholesterol, η^2^ = 0.20, 95% CI [0.00, 0.48]), reflecting the imprecision inherent in the seasonal sample size. A further constraint is that the metabolite (n = 22) and fatty acid (n = 39) analyses were conducted largely on different individuals, with only 11 sharks common to both. Examining the relationship between the metabolites and fatty acids therefore cannot be examined at the individual level, although patterns of inter-individual variation within each dataset are examined. Furthermore, variables such as capture location and capture time may also influence the physiological status of individuals. Future studies should aim to sample white sharks across their entire geographical range and across seasons and multiple years to illuminate changing nutritional and energetic conditions.

## 5. Conclusions

We investigated the energetic state and nutritional condition of juvenile white sharks using a combination of biomarkers. Although aspects of lipid metabolism in large active shark species are not well understood, plasma metabolites, thyroid hormones and fatty acid analyses are useful markers to link energy use/storage and diet. Our findings suggest that the high energetic costs of migration, coupled with the seasonal changes in prey availability and quality in Eastern Australia, may explain the nutritional and energetic variation in white sharks. Lower-quality prey is likely driving this variation since β-hydroxybutyrate concentrations indicate recent feeding. Such knowledge is crucial to improve the currently limited understanding of how environmental factors influence shark foraging and prey selection and provide a link between nutrition and conservation physiology. These results highlight the utility of plasma energy substrates as a valuable tool for conservation physiology in large marine animals.

## Data Availability

The data underlying this article will be shared on reasonable request to the corresponding authors.

## Supplementary Materials

The following supporting information can be downloaded at: https://www.mdpi.com/article/10.3390/biology15151256/s1, Figure S1: Exploratory Principal Component Analysis of fatty acid and metabolite data; Table S1: Pearson correlation matrix values of white shark plasma metabolites; Table S2: Summary of ANOVA results for white shark plasma metabolites across sex and Season; Table S3: Summary of statistical results for white shark plasma fatty acids across sex and season (all seasons).
