# Supplementary material for: Energy Metabolism and Nutritional Condition of Juvenile White Sharks (Carcharodon carcharias; Linnaeus, 1978)
Source: Biology (Basel). 2026 Jul 31;15(15):1256. doi: 10.3390/biology15151256 (PMC13463835; doi:10.3390/biology15151256)
Supplement: Supplementary file 1 [file biology-15-01256-s001.zip › biology-4347082-supplementary.pdf]

Supplementary material

Insight into the energy metabolism and nutritional condition of juvenile white sharks

Rebecca S. Lipscombe, Bianca Rangel De Sousa, Steve Morris, Paul A. Butcher

Table S1. Pearson correlation matrix values of white shark plasma metabolites total protein (TP, grams per litre, g L^{-1}), cholesterol (CHOL, millimoles per litre, mmol L^{-1}), triglycerides (TAG, millimoles per litre, mmol L^{-1}), β-Hydroxybutyrate (BOH, millimoles per litre, mmol L^{-1}) and Thyroxine (T4, nanomoles per litre, nmol L^{-1}).

	**TAG**	**CHOL**	**BOH**	**T_4**	**Water temperature (°C)**
**TP**	-0.28	-0.15	-0.02	-0.16	-0.06
**TAG**	-	**0.62**	-0.36	-0.06	0.15
**CHOL**	-	-	-0.18	-0.14	0.07
**BOH**	-	-	-	0.14	-0.07
**T4**	-	-	-	-	**0.56**

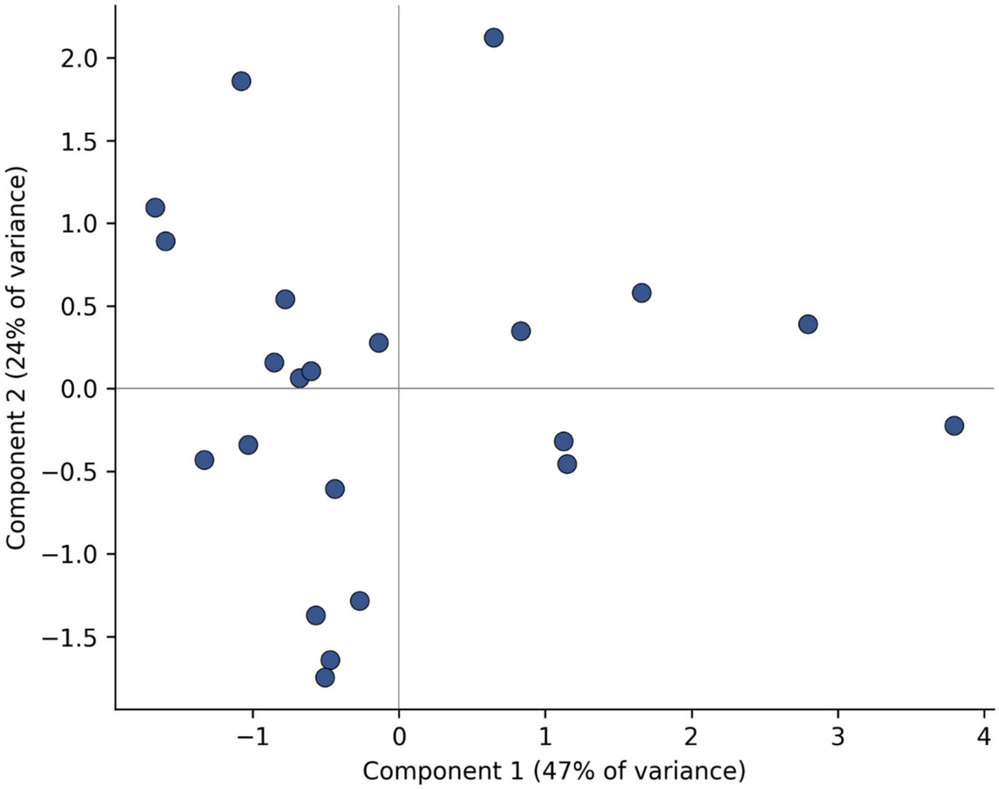

Figure S1. Exploratory Principal Component Analysis of fatty acid and metabolite data.

Table S2. Summary of ANOVA results for white shark plasma metabolites across sex and Season (winter v spring). Significant *P*-values in bold.

**Metabolite**	**Sex**		**Season**	
	$\boldsymbol{F_{(1,22)}}$	***P*-value**	$\boldsymbol{F_{(1,22)}}$	***P*-value**
Total protein g L^{-1}	0.004	0.95	1.07	0.313
Cholesterol mmol L^{-1}	1.28	0.27	0.005	0.95
Triglycerides mmol L^{-1}	0.52	0.48	2.67	0.12
β-Hydroxybutyrate mmol L^{-1}	0.63	0.44	2.10	0.61
Thyroxine nmol L^{--1}	1.00	0.33	0.26	0.16
TAG/CHOL mmol L^{-1}	0.17	0.69	5.07	**0.04**

Table S3. Summary of statistical results for white shark plasma fatty acids across sex and season (all seasons). Tests are one-way ANOVA unless indicated: [k] Kruskal–Wallis test, used where residuals departed from normality. Effect sizes (η^2 with 95% CI) are given for significant seasonal effects. Significant *p*-values in bold.

Fatty acid	Sex statistic	Sex *P*	Season statistic	Season *P*	Tukey's HSD	Season η^2 [95% CI]
**ARA**	H = 0.91[k]	0.34	H = 1.82[k]	0.61	–	–
**DHA**	$F_{1,37}$ = 0.00	0.99	$F_{3,35}$ = 4.45	**0.01**	**Winter–autumn 0.01**	0.28 [0.03, 0.47]
**EPA**	$F_{1,37}$ = 0.61	0.44	$F_{3,35}$ = 0.42	0.96	–	–
**ARA/EPA**	H = 2.99[k]	0.084	$F_{3,35}$ = 0.85	0.48	–	–
**SFA**	$F_{1,37}$ = 0.15	0.69	$F_{3,35}$ = 0.83	0.49	–	–
**PUFA**	H = 0.03[k]	0.86	H = 5.84[k]	0.12	–	–
**PUFAω3**	$F_{1,37}$ = 0.07	0.80	$F_{3,35}$ = 3.70	**0.02**	**Winter–autumn 0.01**	0.25 [0.01, 0.45]
**PUFAω6**	H = 0.32[k]	0.57	H = 2.75[k]	0.43	–	–
**ω3/ω6**	$F_{1,37}$ = 0.16	0.70	$F_{3,35}$ = 3.35	**0.03**	**Winter–autumn 0.03**	0.22 [0.00, 0.42]
